# Adipocyte/breast cancer cell crosstalk in obesity interferes with the anti-proliferative efficacy of tamoxifen

**DOI:** 10.1371/journal.pone.0191571

**Published:** 2018-02-01

**Authors:** Lauriane Bougaret, Laetitia Delort, Hermine Billard, Camille Le Huede, Céline Boby, Anne De la Foye, Adrien Rossary, Ali Mojallal, Odile Damour, Céline Auxenfans, Marie Paule Vasson, Florence Caldefie-Chezet

**Affiliations:** 1 Université Clermont Auvergne, INRA, UNH, Unité de Nutrition Humaine, CRNH Auvergne, F-63000 Clermont-Ferrand, France; 2 Banque de tissus et de cellules, Hôpital Edouard-Herriot, Lyon, France; 3 Plate-Forme d'Exploration du Métabolisme–Composante transcriptomique, Site de Theix, Saint-Genès-Champanelle, France; 4 Service de chirurgie plastique, reconstructrice et esthétique, Hôpital Edouard-Herriot, Lyon, France; 5 Centre anti-cancéreux Jean-Perrin, Clermont Ferrand, France; 6 Cancéropôle Lyon Auvergne Rhône-Alpes (CLARA), Lyon, France; Seconda Universita degli Studi di Napoli, ITALY

## Abstract

**Background:**

Obesity is a well-known risk factor of breast cancer in post-menopausal women that also correlates with a diminished therapeutic response. The influence of adipocytes and their secretome, i.e. adipokines, on the efficacy of hormone therapy has yet to be elucidated.

**Methods:**

We investigated, *ex vivo*, whether mature adipocytes, differentiated from adipose stem cells of normal-weight (MA20) or obese (MA30) women, and their secretions, were able to counteract the effects of tamoxifen (Tx) which is known to decrease neoplastic cell proliferation.

**Results:**

In a tridimensional model and in a model of co-culture, the anti-proliferative effect of Tx on MCF-7 cancer cells was counteracted by MA30. These two models highlighted two different specific gene expression profiles for genes encoding cytokines or involved in angiogenesis based on the adipocyte microenvironment and the treatment. Thus it notably showed altered expression of genes such as *TNFα* that correlated with IL-6. In addition, leptin, IL-6 and TNFα, at concentrations reflecting plasma concentrations in obese patients, decreased the anti-proliferative efficacy of 4-hydroxytamoxifen (a major active metabolite of Tx).

**Conclusions:**

These findings bring insights on adipocytes and mammary cancer cell interactions in Tx therapy, particularly in overweight/obese people. Indeed, patient’ adipokine status would give valuable information for developing individual strategies and avoid resistance to treatment.

## Introduction

Obesity is a risk factor for breast cancer in postmenopausal women [1,2] and an increased BMI is associated with higher risk of metastasis, recurrence and poor final outcome [3–7]. Adipose tissue secretes adipokines, such as leptin, interleukin 6 (IL-6) and Tumor Necrosis Factor α (TNFα), whose plasma concentrations are increased in obese subjects [8–10]. In the breast, adipose tissue and more particularly adipocytes and their secretome, may play a major role in cancer development by surrounding the mammary gland. After menopause, circulating estrogens, which derive from adipose tissue (AT), are associated with an increase of both risk and progression of estrogen receptor positive (ER^+^) breast cancer [5] because estrogens are well known to be involved in breast cancer progression. Indeed, 75% of postmenopausal breast cancer patients develop ER^+^ breast cancer and women presenting ER^+^ breast cancer have poorer final outcome if they are obese compared to women with healthy weight [11]. According to the World Cancer Research Fund, an increase in fat mass of 5 kg/m^2^ among postmenopausal women increases the relative risk (RR) of developing breast cancer (RR = 1.13; 95% confidence interval (CI) = 1.08–1.18) and a weight gain of 10 to 20 kg induces a relative risk of mortality of 1.93 (95% CI = 1.43–2.73).

The higher risk of recurrence and mortality in obese patients could be related to a lesser efficacy of anti-cancer treatments probably due to plasma adipokine variations linked to overweight. Indeed, obese breast cancer women are less sensitive to chemotherapy [12] and present higher mortality rates [13–15]. MDA-MB-231 mammary cancer cells treated with adipose stem cell supernatants present resistance to doxorubicin [16]. By addition, leptin counteracts with the cytotoxic activity of the 5-fluorouracil in colorectal cancer cells [17]. Moreover, we demonstrated that leptin can reduce Tamoxifen (Tx) and chemotherapy efficacy (5-fluorouracil, taxol and vinblastin) in an assessment led on MCF-7 proliferation especially when leptin was used at concentrations reflecting circulating levels found in obese people [18]. Tx, a standard hormone therapy, increases serum leptin levels in postmenopausal breast cancer patients [19,20]. So, leptin may interfere with the efficacy of breast cancer treatments, especially anti-estrogens like Tx that targets ER.

The objective of this research was to evaluate the relationship between obesity and the efficacy of breast cancer treatment with tamoxifen. We evaluated *ex vivo* the impact of adipocyte secretome using human adipocytes from obese and healthy weight women on the efficacy of Tx hormone therapy and focused on specific biomarkers associated with a poor prognosis (leptin, IL-6 and TNFα). This would allow to better understand the risk associated with obesity which could participate to promote therapeutic escape.

## Materials and methods

### Cell culture and reagents

The human breast cancer cell line ERα^+^ MCF-7 and the human breast cells 184B5 from a healthy tissue removed during breast reduction (American Type Culture Collection (ATCC), Molsheim, France), were cultured as previously described [18] according to ATCC recommendations.

Human adipose stem cells (hASCs) were kindly provided by the Cell and Tissue Bank (Hôpital Edouard-Herriot, Lyon, France). hASCs were obtained from patients undergoing surgery for cosmetic purposes without associated pathology according to Helsinki declaration from anonymous healthy donors. Surgical residue was harvested according to French regulation including declaration to research ministry (DC n°2008162) and procurement of written informed consent from the patient. hASCs were extracted from subcutaneous AT from women undergoing optimized liposuction who presented a body mass index (BMI) corresponding to either a normal weight (BMI = 22.4, hASC20), or overweight (BMI = 27.7, hASC27) or obese (BMI = 30.3, hASC30) situations. hASCs were extracted [21] using a 3 mm cannula according to ethical and safety guidelines as approved by the local IRB and as described by Björntorp and differentiated into mature adipocytes (MA) [21].

All the cells used were under *mycoplasma-free* conditions (MycoAlert Plus, mycoplasma detection kit, Lonza, Bale, Switzerland) and cultured in a 5% CO_2_-humidified incubator at 37°C.

### Influence of mature adipocyte secretions on tamoxifen efficacy in a monolayer system

MA obtained after differenciation of hASC [22] from normal (MA20) or obese (MA30) women were cultured (5x10^3^cells/cm^2^, n = 3) for 5 days and conditioned media (CM) collected (CM20 or CM30 respectively). MCF-7 and 184B5 cells were plated in 96-well plates (5x10^3^ cells) and the medium was replaced after 24h by CM20 or CM30 treated or not with Tx (12.5 μM, 72h). Cell proliferation was measured using the resazurin test (Ex_w_ = 530nm and Em_w_ = 590nm, Fluoroskan Ascent FL^®^, Thermo Fisher Scientific, Wilmington, USA).

### Influence of mature adipocyte secretions on tamoxifen efficacy in a co-culture system

Mammary cancer cells were co-cultured with adipocytes (Transwell culture system, porosity 0.4 μm, 5x10^3^ cells/cm^2^; Merck Millipore, Molsheim, France). MCF-7 cells were seeded on the bottom of the system and MA20, MA27 or MA30 on the top chamber. After 24h of co-culture, cells were treated or not with Tx (12.5 μM) and cell proliferation was measured after 72h as described above (n = 3). Mammary and adipose cells cultured alone served as controls.

Mammary breast cells 184B5 (n = 3) were also co-cultured with MA from overweight women (MA27) to assess the role of adipocytes on normal cells.

### Evaluation of cell interactions in 3D model

#### Development of a tridimensional adipose skin equivalent model

Primary cultures of keratinocytes and fibroblasts were established in accordance to ethical and safety guidelines (French regulation n° DC-2008-162) from patients undergoing surgery (child donors, age<10 years), to prepare an adipose skin equivalent model as previously described [22,23].

hASCs (10^5^ cells/skin) from thin and obese women were seeded on the top of a collagen-glycosaminoglycan-chitosan scaffold to constitute a dermal substrate. Fibroblasts (10^5^ cells/skin) were seeded on it to obtain a fatty equivalent dermis after 3 weeks. MCF7 cells or keratinocytes (skin equivalent control) (10^6^ cells) were seeded on the surface and treated or not with Tx (2.5 μM) to obtain 3D adipose skin equivalents (n = 3) [23].

#### Histological analysis

3D adipose skin equivalents were fixed in O.C.T compound (Tissue Teck, Sakura, Netherlands) and frozen (−20°C) [23–25]. Tissue sections were stained with Hematoxylin Phloxin Safran (HPS, Sigma-Aldrich, Saint-Louis, United States) to visualize nucleus, cytoplasm and extracellular matrix formation or with Oil Red O and counterstained with Hematoxylin (Sigma-Aldrich).

#### Evaluation of protein expressions by immunohistochemistry

For immunohistochemical and immunofluorescence staining, Ki67 expression was investigated using affinity-purified polyclonal biotinylated antibodies (Merck Millipore, 1 μg/mL) or monoclonal-mouse primary antibodies to Ki67 respectively (clone MIB1, EnVision, Dakocytomation, Glostrup, Denmark, 1/50). Quantification of proliferative capacity was done by Ki67 positive-cell numeration (n = 3, 3 different lecturers). Nuclear counterstaining using Hoescht was carried out routinely.

#### Evaluation of gene expressions by qRT-PCR

Mammary cells were collected at the surface of the skin after a thermolysin treatment and RNA was extracted with Trizol (Invitrogen, Thermo Fisher Scientific, Waltham, United States). After the evaluation of the quantity and purity (NanoDrop 2000, Thermo Fisher Scientific), DNase treatment (DNase I Amplification grade, Invitrogen) and cDNAs retrotranscription (HighCap cDNA RT Kit RNAse inhib, Invitrogen) were made according to the manufacturer’s recommendations.

Quantitative Real-Time PCR (qPCR) assays were performed on plates designed by Applied Biosystems (TaqMan® Array 96 well Fast Plate, Customformat 48, Part N° 4413257, Lot N°1307140–0001) using SDS7900HT automaton (Applied Biosystems, Thermo Fisher Scientific) with TaqMAN^®^ (Applied Biosystems). The analysis was conducted on 44 genes and 3 references genes with TaqMAN^®^ Array Fast Plates (*18S; UBC; ACTB; LEPR; LEP; ADIPOQ; ADIPOR1; ADIPOR2; AKT1; BAX; BCL2; BRCA1; CCND1; CYP19A1; ESR1; ESR2; IL6; MAPK1; MMP2; MMP9; MYC; PGR; PPARA; PPARG; STAT3; TNF; TP53; GSR; PTGS2; HMOX1; GPX1; GPX4; VEGFA; KDR; THBS1; HIF1A; CDH1; PCNA; ERBB2; AURKA; BIRC5; CCNB1; MYBL2; GRB7; BAG1; MMP11; NME1; CA9*). Actin B (*ACTB*) and Ubiquitin C (*UBC*) were used for normalization. The comparative cycle threshold (CT) method (2^-ΔΔCT^) was used to calculate the relative gene expression of a given sample, normalized within the sample to two reference genes, and relative to the expression of the same gene in another sample: 2^-ΔΔCT^ method with ΔΔCT = [ΔCT (sample1) - ΔCT (sample2)] and ΔCT = [CT(target gene)–geometric mean CT (reference genes)].

### Influence of leptin, IL-6 and TNFα on 4-OH-Tx efficacy in a monolayer system

MCF-7 cells (5x10^3^ cells, 96-well plates, n = 6) were treated or not with leptin (10 ng.mL^-1^, 100 ng.mL^-1^), Il-6 (2.3 pg.mL^-1^, 83 pg.mL^-1^) and TNFα (0.7 pg.mL^-1^, 3.5 pg.mL^-1^) in the presence or not of 4-OH-Tx (12.5 μM). Cell proliferation was measured as described above. The choosen concentrations reflected plasma concentrations in thin or obese women [10,26,27].

### Statistics

Results were expressed as mean +/- SEM. Statistical analysis was performed using the paired, bilateral Student’s t-test with StatView^®^ Software (SAS Institute Inc.) excepted concerning the Ki67 positive-cells (Mann-Withney test).

## Results

### Crosstalk between breast cancer cells and adipocytes or their secretome decreased Tx efficacy on MCF-7 and 184B5 cells, especially in case of obesity

**Fig 1:**
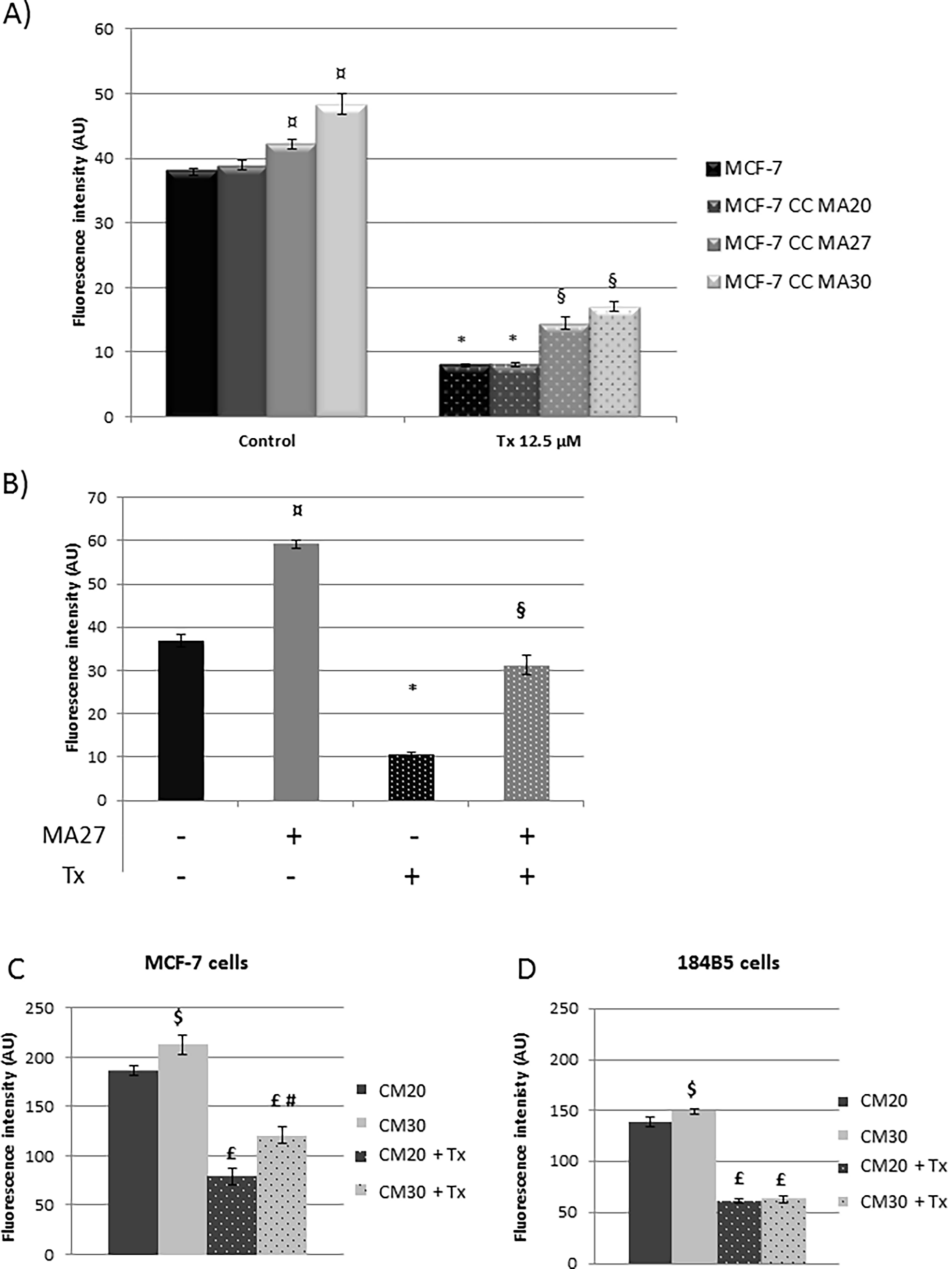
Effect of mature adipocytes, their secretions and Tamoxifen (Tx) on cell proliferation. (A) MCF-7 cells were co-cultured with MA obtained from women of normal weight (MA20), overweight (MA27) or obese (MA30) women and treated with Tx. (B) 184B5 cells were co-cultured with MA27 with or without Tx. For A) and B), the proliferation was quantified after 72h using a resazurine test (Fluoroskan Ascent^®^ FL). (C) MCF-7 and (D) 184B5 cells were also cultured with conditioned media from the culture of MA20 (CM20) and AM30 (CM30) with or without Tx treatment. Results were expressed as Mean ± SEM, ¤ MA *vs* Control, * Tx *vs* Control, § MA+Tx *vs* Tx; $ CM30 *vs* CM20, £ CM+Tx *vs* CM, # CM30+Tx *vs* CM20+Tx (n = 3, Student's t test).

MA27 or MA30 induced a significantly increase of MCF-7 cell proliferation (+15% and 28% respectively, p<0.05) (Fig 1A) and a less efficacy of Tx treatment (-68% or -65% respectively, p<0.05). In 184B5 mammary normal cells, MA increased the proliferation (+61%, p<0.05) (Fig 1B). Tx treatment decreased cell proliferation (-71%, p<0.05), this effect was counteracted with MA (-84%, p<0.05).

Concerning the impact of the secretome, CM30 increased MCF-7 proliferation (+14%, p<0.05) and diminished Tx efficacy compared to CM20 (-43% and -58% respectively, p<0.05) (Fig 1C). Concerning 184B5 cells, CM30 slightly increased their proliferation (+15%, p<0.05) but not affected the antiproliferative effect of Tx (Fig 1D).

### Adipocytes and their secretome decreased Tx efficacy on MCF-7 cells in a tridimensional model and induced variations of gene expressions

**Fig 2:**
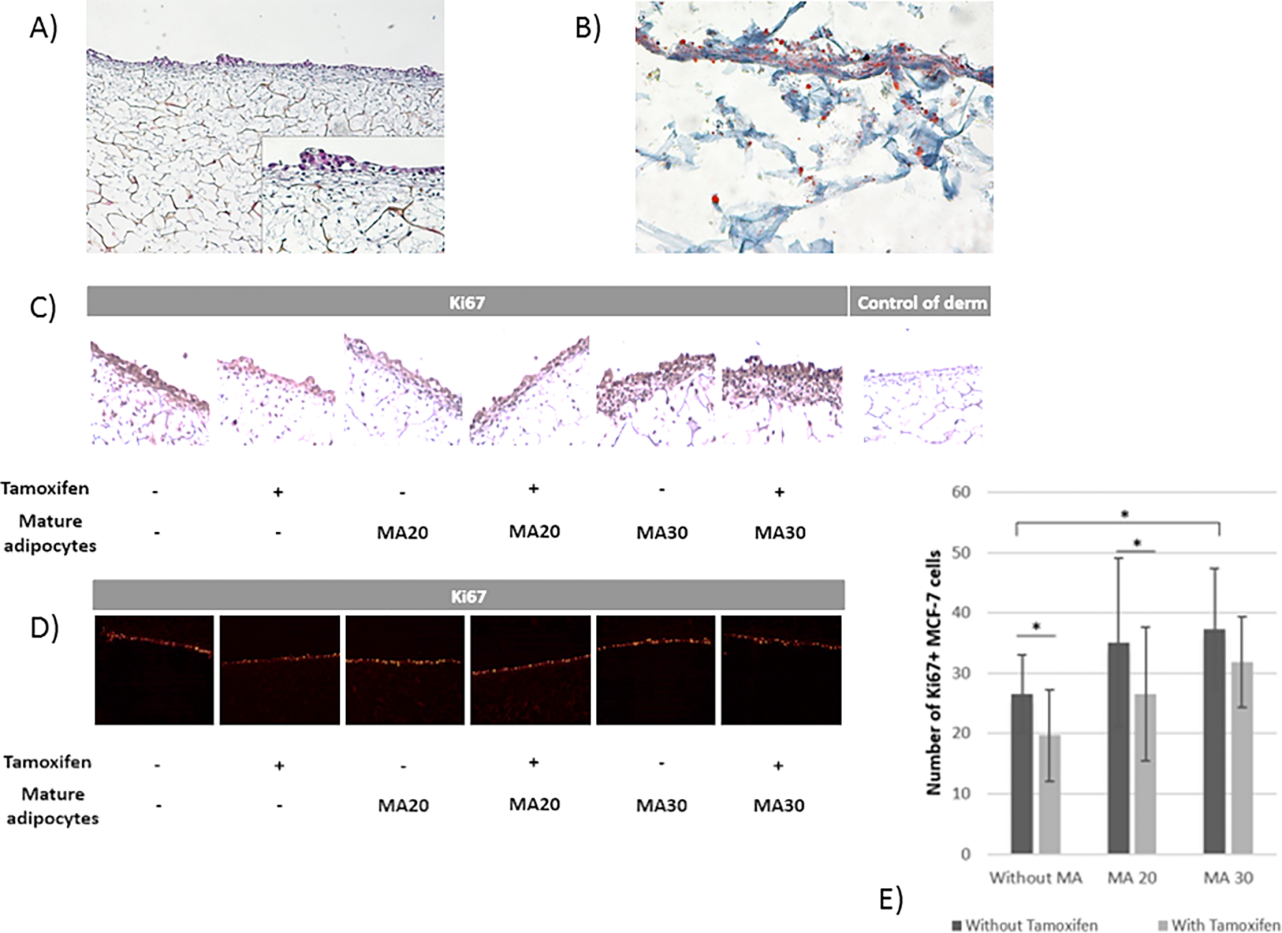
Tridimensional adipose mammary equivalent model. (A) Hematoxylin Phloxin Safran staining showed a pluristratified and differentiated epithelium on a connective underlying tissue made of fibroblasts surrounded by their ECM coloured in yellow in the porous scaffold. (B) Mature adipocytes were visible after Oil Red O staining coloring their vesicles in pink. (C) Ki67 immunohistochemical staining of the tridimensional adipose equivalent model using affinity-purified polyclonal biotinylated antibodies raised against Ki67 (Magnification: ×400). Positive staining appeared in brown. (D) Ki67 immunofluorescent staining of the tridimensional adipose equivalent model. Positive staining appeared in red. (E) Comparison of cellular proliferation in tridimensional adipose equivalent model with or without mature adipocytes and tamoxifen treatment.

The validation of the 3D model was checked by histological analysis as previously described [23]. This analysis was made in order to show the presence of fibroblasts colonizing the porous scaffold surrounded by their extracellular matrix *via* HPS staining (Fig 2A). Oil Red O staining showed the presence of MA differentiated from hASC (Fig 2B).

Without MA, Tx decreased Ki67^+^ MCF7 cell number (Fig 2C). MA30 increased Ki67^+^ MCF7 number but no significant decrease was observed after Tx treatment (Fig 2C, 2D and 2E).

**Fig 3:**
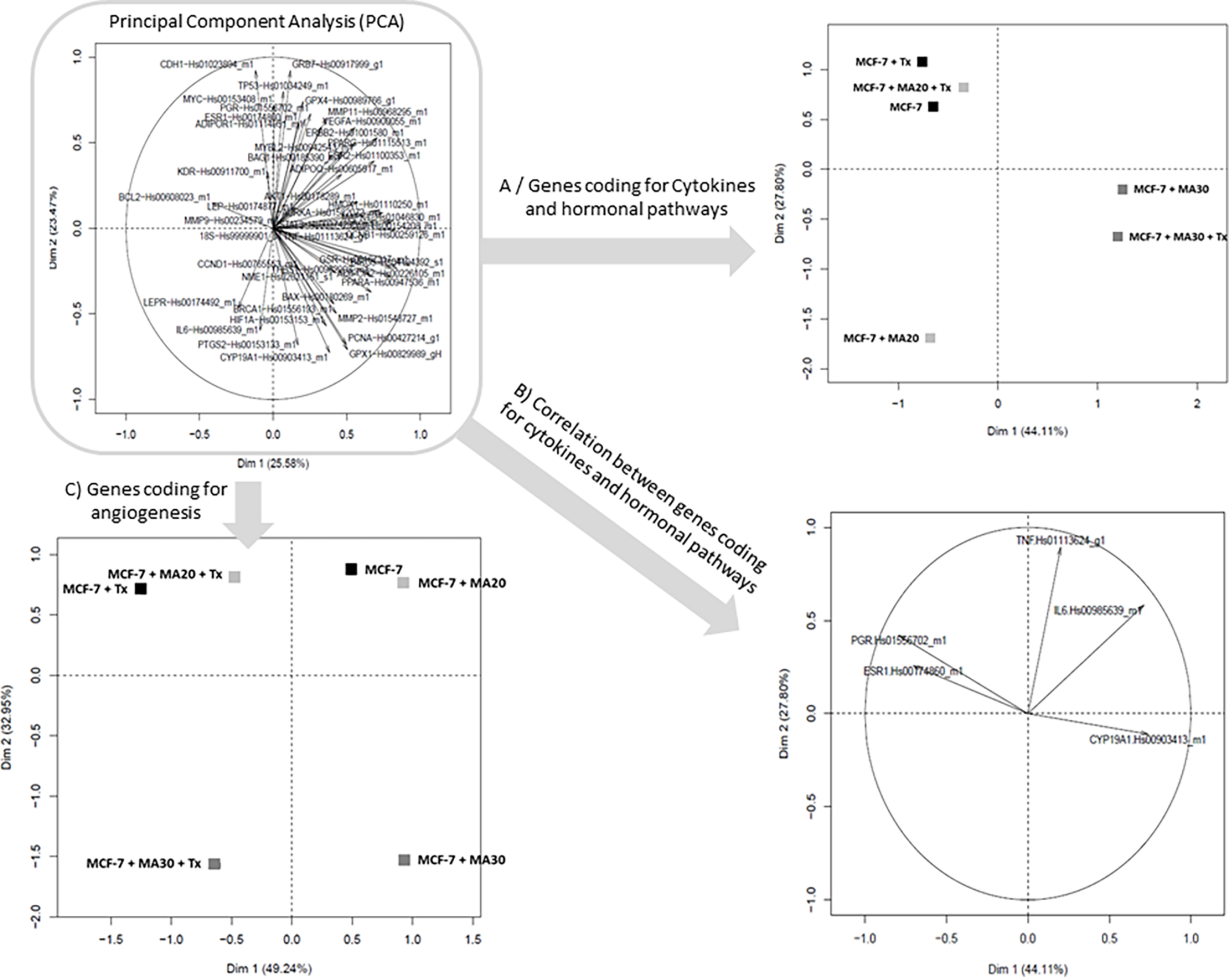
Principal component analysis (PCA) to explore the relationships among genes on 3D adipose skin equivalent model. The expression of genes in MCF-7 mammary cell line co-cultured or not with mature adipocytes from women with a normal weight (MA20) or obese women (MA30) with or without tamoxifen treatment were analyzed by PCA. A first global PCA was carried out (A) followed by a second one taking into account the biological function of the different studied genes (B, C, D, E).

A principal component analysis (PCA) was performed on all the studied genes (Fig 3). In view of the complexity of these results, we decided to carry out this analysis according to the biological functions of the genes eg “adipokines, cytokines and hormonal pathway” (LEP, LEPR, ADIPOQ, ADIPOR1, ADIPOR2, ESR1, ESR2, PGR, IL6, TNF, CYP19A1), “cell cycle and proliferation” (MYC, AKT1, BAX, BCL2, BRCA1, CCND1, MAPK1, PPARA, PPARG, TP53, STAT3, PCNA, ERBB2, AURKA, BIRC5, CCNB1, MYBL2, GRB7, BAG1), “angiogenesis” (CDH1, MMP9, MMP2, VEGFA, KDR, THBS1 HIF1A), “oxidative stress” (GSR, PTGS2, HMOX1, GPX1, GPX4), “treatment response” (MMP11, NME1, CA9, HIF1A, CDH1, PCNA, ERBB2, AURKA, BIRC5, CCND1, MYBL2, GRB7, BAG1).

The restricted PCA analysis concerning “Cytokines and hormonal pathways” (Fig 3A) permitted to segregate cells exposed to an obese environment. In addition, closed correlations have been identified notably between *PGR* and *ESR1* and between *IL-6* and *TNFα* (Fig 3B). When the PCA focused on “angiogenesis”, clusters were obtained according both to an obese environment and to the Tx treatment exposition (Fig 3C).

When the effect of obesity was investigated using expression of specific genes, we observed that *Leptin*, *PGR* and *VEGF* expressions were significantly decreased in MCF-7 co-cultured with MA30 *versus* MCF-7 co-cultured with MA20 (*Leptin*: 0.09-fold, p<0.001; *PGR*: 0.27-fold, p = 0.05; VEGF: 0.48-fold, p<0.05, *vs* MA20) contrary to *TNFα* gene expression which was increased by MA30 (2.71-fold, p = 0.01) (Table 1, column A).

**Table 1:** qRT-PCR assays on 3D adipose equivalent model.

			Column A		Column B		Column C				Column D	
			Obesity effect		Tamoxifen effect		Adipocyte effect with Tx treatment				Obesity effect with Tx	
			MCF7_MA30 / MCF7_MA20		MCF-7_Tx / MCF-7		MCF-7_MA20-Tx/MCF-7_Tx		MCF-7_MA30-Tx/MCF-7_Tx		MCF7_MA30_Tx / MCF7_MA20_Tx	
			R (quantity)	*p* *value*	R (quantity)	*p value*	R (quantity)	*p value*	R (quantity)	*p value*	R (quantity)	*p value*
**Adipokines, cytokines, hormonal pathway**	**LEP**		**0,09**	**0,00001**	1,00	0,99	1,13	0,68	0,49	0,04	**0,44**	**0,02**
	**TNF**		**2,71**	**0,01**	1,07	0,83	1,02	0,96	0,65	0,20	0,64	0,18
	**PGR**		**0,27**	**0,05**	1,26	0,70	0,77	0,68	**0,23**	**0,03**	0,30	0,07
	**ESR1**		0,59	0,16	0,68	0,30	0,57	0,14	0,48	0,06	0,84	0,64
	**IL6**		1,34	0,50	1,25	0,61	0,99	0,98	1,02	0,97	1,03	0,95
	**CYP19A1**		1,34	0,93	0,57	0,25	0,79	0,62	1,62	0,33	2,05	0,16
	**ADIPOR1**		1,05	0,87	0,95	0,84	0,72	0,25	0,65	0,14	0,91	0,73
	**ADIPOR2**		0,77	0,87	0,84	0,55	0,89	0,70	0,83	0,53	0,93	0,81
	**PPARA**		0,84	0,61	0,72	0,34	1,13	0,71	0,92	0,80	0,81	0,54
	**PPARG**		0,74	0,32	0,79	0,43	0,93	0,79	0,87	0,63	0,94	0,79
**cell cycle and proliferation, oncogene**	**BRCA1**		0,77	0,49	**0,40**	**0,03**	1,35	0,43	0,94	0,86	1,35	0,82
	**CCNB1**		1,12	0,73	0,63	0,17	**0,50**	**0,05**	0,74	0,36	1,50	0,23
	**MYBL2**		1,08	0,76	0,77	0,33	0,63	0,10	0,81	0,43	1,29	0,34
	**ERBB2**		0,56	0,09	1,39	0,30	0,58	0,10	0,66	0,19	1,14	0,68
	**CCND1**		0,71	0,18	0,90	0,68	1,06	0,80	1,21	0,43	1,14	0,59
	**STAT3**		0,84	0,51	0,98	0,95	**0,55**	**0,04**	0,85	0,54	1,54	0,11
	**MAPK1**		0,91	0,74	0,78	0,38	0,77	0,35	1,06	0,83	1,39	0,25
	**TP53**		1,04	0,89	0,92	0,78	0,88	0,66	0,87	0,65	0,99	0,98
	**AKT1**		1,04	0,87	0,97	0,89	0,83	0,41	1,02	0,93	1,23	0,36
	**BAX**		1,04	0,89	0,81	0,47	1,05	0,86	1,31	0,36	1,24	0,45
	**BIRC5**		1,34	0,53	0,56	0,23	0,66	0,38	1,04	0,93	1,59	0,33
**Angiogenesis**	**VEGFA**		**0,48**	**0,03**	1,01	0,98	0,81	0,50	**0,41**	**0,01**	**0,5**	**0,1**
	**HIF1A**		0,67	0,32	**0,42**	**0,05**	1,79	0,16	1,13	0,75	0,63	0,27
	**THBS1**		1,04	0,89	0,80	0,67	1,21	0,72	0,98	0,97	0,81	0,69
	**MMP2**		1,31	0,31	0,85	0,53	0,95	0,83	1,31	0,31	1,38	0,23
**Oxidative stress**	**HMOX1**		0,61	0,13	0,81	0,49	**0,51**	**0,1**	0,81	0,50	1,58	0,16
	**GSR**		0,80	0,41	0,83	0,48	0,90	0,69	0,95	0,85	1,06	0,83
	**GPX1**		0,78	0,60	0,80	0,64	1,08	0,87	1,71	0,26	1,58	0,33
	**GPX4**		1,30	0,39	1,02	0,95	0,80	0,46	0,75	0,35	0,94	0,84
	**PTGS2**		0,69	0,47	0,75	0,57	1,44	0,48	1,05	0,93	0,73	0,54
**Treatment response**	**CDH1**		1,13	0,82	1,25	0,67	0,46	0,17	0,41	0,11	0,89	0,82
	**CA9**		0,65	0,16	0,82	0,51	0,71	0,25	0,92	0,79	1,30	0,37
	**NME1**		0,54	0,14	0,92	0,83	1,15	0,73	0,80	0,58	0,70	0,38
	**AURKA**		1,02	0,94	0,86	0,58	0,60	0,08	0,58	0,07	0,97	0,91
	**GRB7**		0,89	0,80	1,95	0,16	0,45	0,09	**0,36**	**0,04**	0,80	0,62

qRT-PCR analysis was conducted on MCF-7 cells co-cultured with MA from normal (MA20) or obese (MA30) women with or without tamoxifen treatment (TaqMAN® (Applied Biosystems)). *ACTB* and *UBC* were used for normalization. The comparative cycle threshold (CT) method (2-ddCT) was used to calculate the relative gene expression of a given sample, normalized within the sample to two endogenous reference genes. The results are significant when p value is inferior to 0.05 (indicated in the table).

When cells were treated with Tx, *Leptin* expression remained unchanged whereas a significant decrease of *BRCA1* and *HIF1A* expression was observed (Table 1, column B).

When adipose microenvironment was evaluated on Tx efficacy (MCF-7 co-cultured with both adipocytes and tamoxifen versus MCF-7 cultured only with Tx) (Table 1, column C), *Leptin* expression was unchanged in comparison with MCF-7 cultured only with Tx. A decrease of *HMOX1* was observed for MCF-7 co-cultured with MA20 (R = 0.51, p = 0.05, *vs* without MA) whereas a decrease of *VEGFA*, *PGR* and *GRB7* was observed for MCF-7 with MA30 (R = 0.41, p = 0.01; R = 0.23, p<0.05; R = 0.36, p<0.05 respectively, *vs* without MA).

When we investigated the impact of both obesity and Tx treatment, *Leptin* and *VEGF* expressions were decreased in MCF-7 cells co-cultured with MA30 and Tx (R = 0.44, p<0.05; R = 0.51, p = 0.05, *vs* co-cultured with MA20 and Tx). These results confirmed the results obtained in the 3D system.

Other gene expressions like *ESR1*, *IL-6*, *CYP19A1*, *AdipoR1*, *AdipoR2*, *PPARα and PPARγ* were not altered by MA or Tx treatment.

### Leptin, IL-6 and TNFα decreased 4-OH-Tx efficacy on neoplasic MCF-7 cells

**Fig 4:**
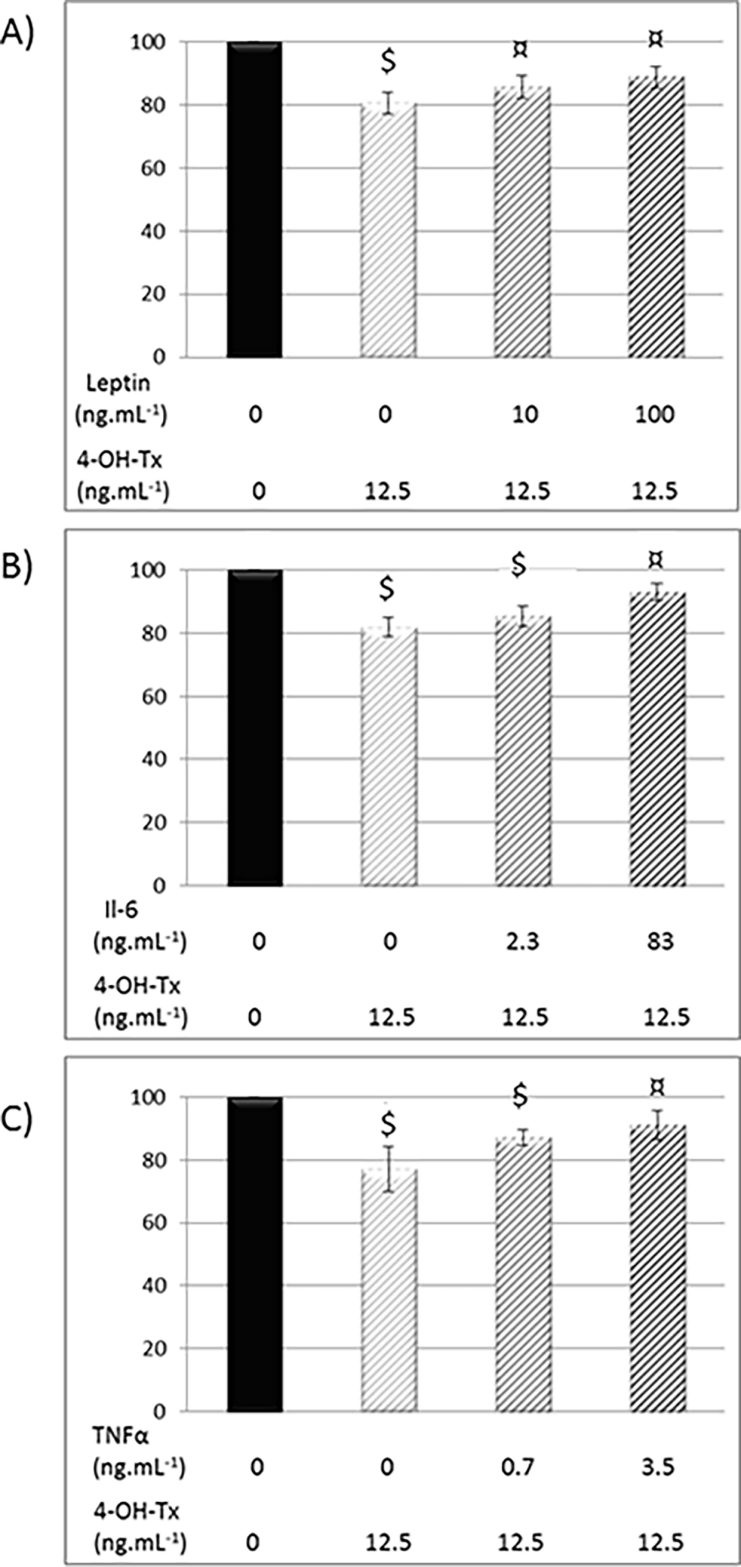
Effects of Leptin, IL-6 and TNFα on MCF-7 cell proliferation after 4-OH-tamoxifen treatment. After adhesion, MCF-7 were treated with leptin (0; 10; 100 ng/mL) (A), IL-6 (0, 2.3; 83 pg/mL) (B), TNFα (0; 0.7; 12.5 pg/mL) (C) and with 12.5 μM of 4-OH-Tx. After 72h, cell proliferation was quantified using a resazurine test (Fluoroskan Ascent^®^ FL). Mean ± SEM, $ 4-OH-Tx +/- adipokine *vs* Control, ¤ 4-OH-Tx +/- adipokine *vs* 4-OH-Tx (n = 3, Student's t test).

We previously showed that leptin (100 and 1,000 ng/ml) increased MCF7 proliferation and reduced Tx efficacy [18]. In the present study, we demonstated that the presence of leptin, IL-6 and TNFα, used at concentrations reflected plasmatic levels in obese people, also diminished the efficacy of 4-OH-Tx, an active metabolite of Tx (-11%, -7%, -9% respectively) (Fig 4).

## Discussion

Obesity is a risk factor for breast cancer development in postmenopausal women and increases metastasis and recurrence associated with changes in serum adipokines [1,3,6]. A lesser therapeutic response is always described for obese patients and could be related to obesity or change in body weight. Indeed, an increase of relapses or mortality is described in obese women receiving chemotherapy [13,28]. An hyperleptinemia is also described in women treated by Tx [19]. In our paper, the relationship between obesity, adipokines and Tx therapy was investigated by evaluating interactions between cancer and adipose cells, and the specific role of adipocyte secretome. The strength of our study is to use *ex-vivo* human mature adipocytes from women of normal weight, overweight and obese women.

With the model of co-culture between MA and mammary cells, we brought the evidence of the *role of MA in breast cancer growth*. In our results, women MA obtained after hASCs differentiation were able, *ex vivo*, to significantly increase the proliferation of both mammary cancer cells (MCF-7) and normal mammary cells (184B5). Co-culture experiments between MA isolated from rat subcutaneous adipose tissue have also demonstrated their ability to stimulate the proliferation of breast cancer cell lines (MCF-7, T47D cells…) [29]. It was also shown that adipose tissue co-cultured with rat mammary tumor cells (CRL1743 cells) resulted in an increase in growth and migration of tumor cells [30].

To highlight the *influence of adipocyte secretome on Tx efficacy*, we used conditioned media obtained from the culture of MA20 or MA30 (CM20 and CM30) and showed that CM30 raised MCF-7 and 184B5 proliferation compared to CM20 treatment. Similarly, the effects of adipocyte differentiation on proliferation and migration of normal (NMuMG) and tumoral (LM3) murine breast epithelial cells were increased with 3T3-L1 conditioned media [31]. In studies using human samples, the incubation of normal (MCF10a) and malignant (MCF-10CA1) breast epithelial cells with breast adipocyte conditioned media (Adip-CM) increased cell motility [32].

Until now, only few studies focus on the relationship between human mammary and adipose cells in case of obesity. So, we investigated obesity effect using a co-culture model between MCF-7 and MA obtained from normal weight (MA20), overweight (MA27) and obese (MA30) women on Tx efficacy, to bring out the influence of the dialogue between these two cell types on treatment resistance. To the best of our knowledge, our study is the first to report an *ex vivo* impact of adipocytes obtained from obese women compared to women of normal weight.

We showed that human MA, differentiated from hASCs, were able to induce MCF-7 proliferation by increasing the number of Ki67 positive-cells. This effect was more pronounced with MA27 and MA30. A recent study using hASC, derived from the abdominal subcutaneous AT of obese subject (BMI>30), has shown an enhanced breast cancer cell proliferation (MCF-7 and MDA-MB-231 cells) and tumorogenesis in immunodeficient mice [33]. As expected, we observed that Tx treatment was efficient to reduce the MCF-7 cell proliferation, whereas its effect was significantly counteracted in the presence of MA27 or MA30. Our results could be correlated with the breast cancer cell (SUM159PT) radioresistance observed when MA obtained from 3T3 murine preadipocyte differentiation were added [34].

To highlight the *impact of cell-cell interaction and obesity* in a more physiological model, we developed an original 3D model [23], wich mimic breast tumor in contact with a connective tissue containing AT assessing the interactions between the different cell types in the presence or not of Tx treatment. Similarly to monolayer co-culture, the adipose microenvironment from obese women induced a higher proliferation of breast cancer cells. Tx treatment decreased MCF-7 proliferation and interestingly the presence of MA from obese women reduced its efficacy. A gene expression analysis permitted us to classify genes according to their biological functions such as “angiogenesis process”, “cytokines and hormonal pathways”. The PCA highlighted two groups from the panel of genes according to adipocyte microenvironment and Tx treatment. Considering “cytokines and hormone” gene expression, only a positive correlation between *PGR* and *ESR1* may be identified independently of adipocyte microenvironment and Tx treatment. In addition, in this 3D model, the expression of *TNFα* was increased, in case of obesity, and a positive correlation was highlighted between genes coding for inflammatory proteins such as IL-6 and TNFα. Indeed, TNFα was demonstrated as being able to regulate the expression of other cytokines such as IL-6 [35]. It was consistent with our results because, i) an increase of TNFα expression was found in MCF-7 cells after co-culture with MA30, compared to MA20; ii) analysis of conditioned media of co-culture between MCF-7 and MA30 compared to those of MCF-7 showed that IL-6 secretion was increased.

To investigate further which adipokine can be involved in this effect, we focused on leptin, IL-6 and TNFα. We previously described that leptin decreases Tx efficacy [18] and we used in the present study the 4-OH-Tx, a Tx active metabolite. Leptin, IL-6 and TNFα decreased the anti-proliferative effect of 4-OH-Tx on MCF7 cells. This crosstalk between leptin, IL-6 and TNFα may be considered since for example, epithelial ovarian cancer which presenting an autocrine production of TNFα have greater release of IL-6 [36]. Moreover, the stimulatory effect of adipose stromal cells on migration and invasion of breast tumor cells is abrogated by a depletion of IL-6 [37].

Concerning normal mammary cells 184B5 cells, the decrease of proliferation could be due to another Tx target since it was demonstrated that ligands which bind to antiestrogen-binding site (AEBS) could inhibit cell proliferation in a dose dependent manner [38]. Another hypothesis was that Tx could induce Protein Kinase C inhibition, which resulted in oxidative stress, and was followed by an inhibition of proliferation [39].

## Conclusions

Our data showed that adipocyte secretome may reduce the efficacy of Tx therapy in case of overweight/obesity. Further studies are therefore needed to better understand the precise role played by adipose tumor microenvironment and by adipokines such as IL-6, leptin and TNFα in breast cancer progression. Indeed, the identification of specific biomarkers may allow a personalized management of overweight breast cancer patients.

## Abbreviations

BMI: Body Mass Index
MA20: Mature adipocytes differentiated from stem cells of normal-weight (BMI = 20) women
MA27: Mature adipocytes differentiated from stem cells of overweight (BMI = 27) women
MA30: Mature adipocytes differentiated from stem cells of obese (BMI = 30) women
